# Computed Tomography-Based Characterization of the Fatigue Behavior and Damage Development of Extruded Profiles Made from Recycled AW6060 Aluminum Chips

**DOI:** 10.3390/ma12152372

**Published:** 2019-07-25

**Authors:** Alexander Koch, Philipp Wittke, Frank Walther

**Affiliations:** Department of Materials Test Engineering (WPT), TU Dortmund University, Baroper Str. 303, D-44227 Dortmund, Germany

**Keywords:** hot extrusion, fatigue development, aluminum chip solid state recycling, intermittent computed tomography, alternating current potential drop (ACPD)

## Abstract

The possibility of producing profiles directly by hot extrusion of aluminum chips, normally considered as scrap, is a promising alternative to the energy-intensive remelting process. It has to be taken into account that the mechanical properties depend on the quality of the weld seams between the chips, which arise during the extrusion process. To estimate the influence of the weld seams, quasistatic and cyclic investigations were performed on chip-based profiles and finally compared with cast-based extruded profiles. In order to gain comprehensive information about the fatigue progress, different measurement techniques like alternating current potential drop (ACPD)-technique, hysteresis measurements, and temperature measurements were used during the fatigue tests. The weld seams and voids were investigated using computed tomography and metallographic techniques. Results show that quasistatic properties of chip-based specimens are only reduced by about 5%, whereas the lifetime is reduced by about a decade. The development of the fatigue cracks, which propagate between the chip boundaries, was characterized by an intermittent testing strategy, where an initiation of two separate cracks was observed.

## 1. Introduction

Because of the increasing scarcity of resources, demands with regard to lightweight construction have significantly increased in recent years [1]. In this context, aluminum is particularly suitable because of the excellent strength-to-weight ratio and is becoming more and more popular in lightweight-relevant fields such as the automotive and aerospace industries [2]. A disadvantage is the energy-intensive production of primary aluminum compared to other construction metals [3,4]. With a requirement of about 200 GJ per ton, the production of primary aluminum is one of the most energy-intensive production processes [3] and thus exceeds steel production by a factor of ten [4]. To reduce this large amount of energy, more use is being made of secondary aluminum. Conventionally, the aluminum is melted for recycling [5]. A promising alternative with significantly lower energy consumption is solid state recycling by hot extrusion, in which aluminum scrap can be formed directly into profiles. Compared to the remelting process, direct recycling enables a reduction of energy up to 31% [6]. The use of such scrap like chips additionally has the distinct advantage of a reduced price compared to raw aluminum as well as a less material loss due to the high demand of oxides on the surface of the chips [6]. Stern developed the procedure in 1944 [7], albeit the process has only been intensively studied in the last two decades [8,9,10].

Regarding the mechanical properties of chip-based profiles, it is questionable to what extent the profiles produced by this way meet the requirements in terms of strength and durability. As previous studies show [11,12], the mechanical properties of chip-based profiles depend on the quality of the weld seams occurring between the chips during the extrusion process. The bond strength of the welded aluminum chips mainly depends on time of the contact of the chips and the temperature, as diffusion was found out to play a significant role in the bonding mechanism [12]. On the one hand, the oxide layers have to be broken up in order to enable a direct metal-to-metal contact. On the other hand, the distance where the surfaces are in contact has to be long enough in order to transfer enough energy for the diffusion process, which will be strongly activated by high pressure and temperature. As Cooper and Allwood [13] showed, the influence of the temperature cannot only be explained by the influence on the flow stress of the aluminum, but also the high dependence of the diffusion on the temperature. Because of this, below room temperature process time does not have a significant influence on the bond strength, as diffusion is not prevalent for such temperatures [13].

In the case of a hot extrusion process parameters such as shear stress, pressure, and local strain during the extrusion are critical for a sufficient break-up of the oxide layers and therefore a satisfactory diffusion and welding process [11,12,14]. These parameters can be adjusted by process parameters, especially the extrusion ratio, the ram speed, and the die design [11].

Most known methods to achieve the required process parameters are attributed to the SPD (severe plastic deformation) method [15], which realizes the break-up of the oxides via high local strains and high hydrostatic pressures. While hot extrusion process has often been used to realize the high local strain [11,14,16], other methods have also been investigated such as friction stir extrusion processes [17,18], which have undergone a change in microstructure and hot and cold cracking. Approaches using a compression process at room temperature [9,19] did not lead to success because the shearing forces were too low. Instead, an additional forming process, such as a rolling process [20] was necessary. To significantly increase the local strains, ECAP (equal channel angular pressing) processes were also used which significantly increase the ductility of the resulting profiles and cause grain refining [21]. However, a disadvantage is a more complex process control and significantly increased forces.

Previous investigations [11,12] particularly address the quasistatic properties of chip-based profiles. First investigations regarding cyclic properties [22,23] show a crack propagation along the chip boundaries so that these act as weak links. This leads to a reduction of the lifetime up to a factor of ten [22,23]. A summary of studies regarding different methods of solid state recycling is given by [21], whereby [15] summarizes recent results of mechanical investigations as well as influencing parameters.

The aim of this study is therefore to investigate the mechanisms that lead to the reduced lifetime observed in [22]. In this context, quasistatic and cyclic investigations are used to identify the possible parameters influencing the mechanical properties of extruded chip-based profiles. The focus lies on constant amplitude tests, with stress amplitudes, estimated by load increase tests, which are described in [24]. All specimens were analyzed by X-ray computed tomography (CT) before the tests in order to be able to detect possible influences of defects like pores and delaminations on the mechanical properties.

In order to draw conclusions on the damage development under constant as well as variable amplitude load, supportive measurands, such as the plastic strain amplitude, the change in temperature and the change in alternating current (AC) potential were used. In this context, the alternating current potential drop (ACPD)-technology is the most comprehensive, as it depends on the temperature, the geometry of the specimen, the microstructure, and the defect structure [24,25]. Based on the measured material response, time-dependent microstructural processes that lead to fatigue fracture can be followed.

In order to be able to comprehensively analyze the development of damage during cyclic loading, intermittent fatigue tests were carried out. Therefore, fatigue tests were interrupted at certain numbers of cycles with specific material reactions and characterized by computed tomography in order to determine the crack propagation.

## 2. Experimental Methodology

### 2.1. Material and Process Route

In order to characterize the quasistatic and cyclic behavior of directly recycled profiles, chips made of EN AW6060 aluminum alloy were used as a basis for the experiments. The chemical composition was determined by Otto Fuchs Dülken GmbH (Viersen, Germany) by X-ray fluorescence analysis (XRF) and is given in Table 1. The values determined are within the specified limits according to standard (DIN EN 573-3).

**Figure 1:**
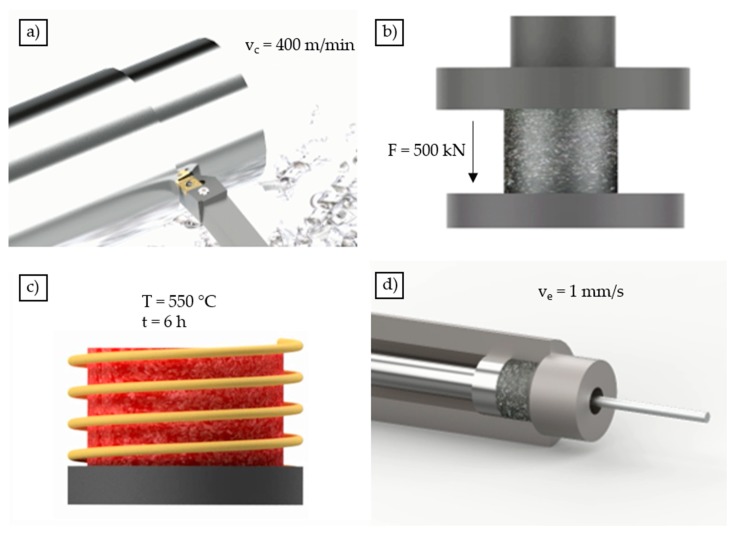
Schematic representation of the extrusion process steps: Machining of the chips (**a**), pre-compaction (**b**), heat treatment (**c**), hot extrusion (**d**).

In this study, the usability of chips for extrusion and the effects on the mechanical properties of chip-based specimens were investigated and compared with conventional cast-based material. To this end, the cast-based and chip-based material were hot extruded with the same flat-face die for comparison purposes. The process route for the production of the chip-based profiles consists of four stages and is shown in Figure 1.

To produce the extruded profiles, the chips were first produced using AW6060 cast bars by a machining process at the Institute of Machining Technology (ISF) at TU Dortmund University. The geometry of the chips was modified based on relevant research work of Haase et al. [14] and Güley et al. [11] in such a way that they can be expected to have best welding properties resulting in best mechanical properties. The spirally shaped chips have a length of l_c_ = 11.0 ± 1.7 mm, a width of w_c_ = 7.6 ± 1.2 mm, and a thickness of t_c_ = 1.1 ± 0.4 mm [14]. For the machining of the chips, a cutting insert made by Sandvik (VBMT 160404-UR4225) was used and the chips were produced using a cutting speed of v_c_ = 400 m/min, a feed rate of f_c_ = 0.4 mm, and a cutting depth of a_p_ = 2.25 mm [14].

To ensure enhanced chip surfaces, the chips were cleaned from machining lubricant as well as contaminants and dried after production. In order to ensure satisfactory welding of the chips in the extrusion process, the chips were pre-compacted by a single stroke process at room temperature, using a compaction force of F = 500 kN. Finally, the compacted blocks, with a mass of m_B_ = 550 g, a length of l_B_ = 92 mm, a diameter of d_B_ = 60 mm, and a relative density of 78% were homogenized for 6 h at 550 °C, preheated to reduce the necessary extrusion force and then extruded. In order to ensure comparable circumstances, the cast-based material underwent the same heat treatment (homogenization) and the same extrusion parameters as the chip-based billets.

The individual extrusion process parameters were also investigated in detail in [11,14] and optimized with regard to the resulting properties of the extruded profiles. Therefore, the blocks were heated up to a temperature of T_B_ = 550 °C and were extruded with a ram speed of v_e_ = 1 mm/s using a Collin LPA250t hydraulic extrusion press with a maximum ram force of 2.5 MN. The tool was heated up to a temperature of T_T_ = 450 °C. The flat-face die used for the extrusion process had a diameter of d_d_ = 12 mm which results in an extrusion ratio of R_p_ = 30.25. The extrusion ratio is defined as the quotient between the diameter of the billet (d_B_ = 66 mm) and the diameter of the resulting profile (d_d_ = 12 mm). The extruded profiles were cooled by compressed air after leaving the die.

### 2.2. Metallography

In order to be able to correlate the microstructure with the mechanical properties, a more precise knowledge of the chip orientation and the grain structure, which directly affects the strength according to the Hall-Petch relationship [26], is of importance. Therefore, cast-based and chip-based profiles were cut and cold-embedded perpendicular to the extrusion direction. The profiles were then ground and polished up to a grit size of 0.1 µm using SiO_2_ polishing suspension. The microstructure was characterized on cross-sections by means of an electrolytic etching according to Barker [27]. Fluorophosphoric acid (35%) was used as an electrolyte at a flow rate of 12 L/min. On the profile, poled as the anode, a layer applied by the etching process which enables the detection of the grain orientation. The etching was carried out for 90 s at a DC voltage of 20 V using an electrolytic etching device (Struers LectroPol-5, Willich, Germany). The subsequent microstructural characterization under polarized light was carried out on a light microscope (Zeiss Axio Imager M1m, Jena, Germany). Subsequently, the grain size was determined by the linear intercept method.

### 2.3. Fractography

In order to be able to characterize in particular the deformation and crack propagation behavior of the specimens, the fracture surfaces of the tested specimens were examined in a scanning electron microscope (SEM) (Tescan Mira 3 XMU, Brno, Czech Republic). For a comprehensive characterization of the fracture surfaces, both the information of the element-sensitive backscattered electron detector and the secondary electron detector suitable for topological information were evaluated. Previously, the fracture surfaces were cleaned in an ethanol-filled ultrasonic bath. The investigations were intended to detect stress-dependent changes in the type, shape, and size of cracks and to determine differences in the fatigue behavior between the cast- and chip-based specimens. For the chip-based specimens, knowledge about the preferred crack propagation direction and the role of the welded chips in the fatigue process, as well as their interaction, was gained.

### 2.4. Mechanical Testing

#### 2.4.1. Tensile Tests

All tensile tests were carried out strain-controlled according to DIN EN ISO 6892-1 at room temperature on a universal testing machine (Instron 3369, High Wycombe, UK) equipped with a load cell with a maximum force of 50 kN. After the machining process, the specimens had a roughness of R_z_ = 25 μm in the gage length area. Before the tests, the gage length areas of the specimens were ground by means of abrasive paper and then polished using polishing paste.

**Figure 2:**
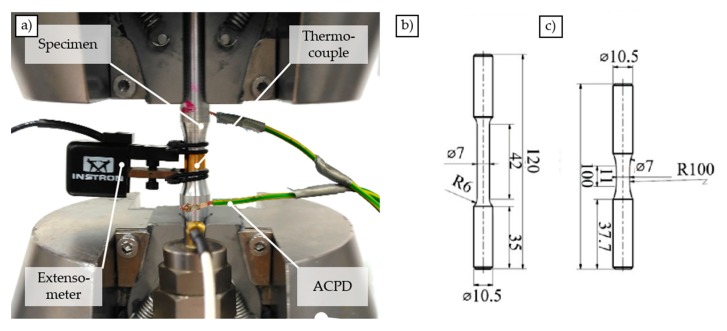
Experimental setup for fatigue experiments (**a**), specimen geometry for quasistatic (**b**), and fatigue (**c**) tests, all units in mm.

For strain measurement, a tactile extensometer (Instron type 2630-106) with a gage length of 25 mm and a maximum extension of +50%, −10% was used. The specimen geometry, according to DIN 50125 (tensile test DIN 50125-A 7 × 35) is shown in Figure 2b. The tests were carried out using a strain rate of 0.00025 s^−1^ in the elastic and 0.001 s^−1^ in the elastic-plastic range. The transition to the elastic-plastic range was assumed when exceeding a normal stress of 50 MPa.

#### 2.4.2. Fatigue Tests

Various fatigue tests were carried out in order to determine the load-dependent deformation and damage behavior. Therefore, continuous load increase tests were carried out, described in [24] as a basis to estimate suitable stress amplitudes for the constant amplitude tests. The geometry of the specimens used for this purpose is shown schematically in Figure 2c. All fatigue tests were carried out on a servohydraulic testing machine (Instron 8872) with a load cell with 10 kN load capacity. The tests were performed without superimposed mean stress with a stress ratio of R = −1, a test frequency of f = 10 Hz, and a sinusoidal load–time curve. Specimens, which exceeded N_l_ = 2 × 10^6^ load cycles were defined as run outs.

In order to follow the material reactions, the characteristics of the stress–strain hysteresis were detected by means of a tactile extensometer (Instron type 2620-603, l_0_ = 10 mm), the change in electrical resistance by using ACPD (alternating current potential drop) technique, as well as the deformation and damage induced change in temperature by means of thermocouples. In addition to the thermocouple attached to the specimen, the ambient temperature was recorded by three thermocouples placed at different areas in the vicinity of the specimen. As a variable room temperature, which is included in the calculation of the temperature changes of the specimen, the mean value of the temperature measurements of these three additional thermocouples was used. To measure the microstructure sensitive change in AC (alternating current) potential, Matelect CGM-5 system (Harefield, UK) was used. The electrical contacts were spot welded to the specimens, while the poles of the current introduction and the poles of the measurement of the potential were welded each crosswise to reduce interference effects. The current was kept constant at a value of I = 1.7 A, with a signal gain of 90 dB. The frequency f_AC_ was found out to be optimal at a value of f_AC_ = 0.3 kHz. The experimental setup is shown in Figure 2a.

### 2.5. Computed Tomography-Based Defect Analyses

For the analyses of the internal defect structure as well as the defect development of the cast- and chip-based specimens under cyclic loading, computed tomography (CT) examinations were performed using Nikon XT H 160 X-ray computed tomography scanner (Leuven, Belgium). In order to correlate the defect characteristics as well as the defect distribution with the quasistatic and cyclic properties, all specimens were examined by CT before testing. The volume reconstructions and defect analyses of the CT scans were realized using VGStudio Max 2.2 software. To ensure the comparability of the results of the defect analyses, all specimens were investigated with the same scanning parameters. The parameters which were found to be optimal with regard to the expected image quality are summarized in Table 2.

In addition to the defect analyses, investigations of the damage development of the extruded chip-based specimens were tracked intermittently. For this purpose, a chip-based specimen was loaded with a certain number of load cycles and then analyzed by CT. Doing so, changes in the internal defect structure, as well as preferred crack initiation sites and propagation direction, were characterized. The fatigue test was each interrupted when significant changes in the material reactions occurred.

## 3. Results and Discussion

### 3.1. Metallographic Investigations

**Figure 3:**
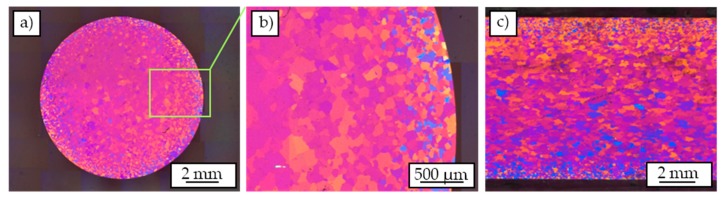
Micrographs of Barker-etched cast-based profile: overview of cross-section (**a**), detailed view of cross-section (**b**), and overview of longitudinal section (**c**).

After barker etching, the cross-section of the cast-based profile (Figure 3a,b) shows an inhomogeneous grain size distribution in radial direction. In the marginal areas of the cast-based profile significantly smaller grains can be recognized, whereas the grains in the middle of the profile are much larger. Overall, the grains in all areas show a round and uniform shape.

The optical micrograph of the longitudinal section (Figure 3c) also shows the described inhomogeneity with respect to the grain size distribution and a round shape. The mean grain size in the center of the profile was determined by means of the linear intercept method to be 380 ± 58 μm and 82 ± 37 μm in the marginal area.

**Figure 4:**
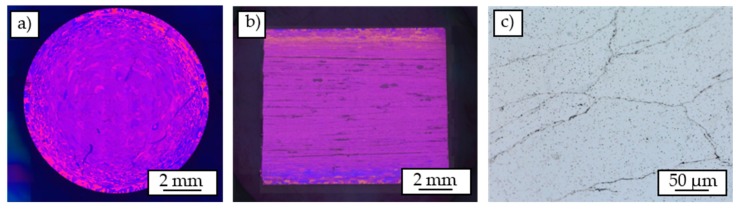
Overview of Barker-etched cross-section (**a**), Barker-etched longitudinal section (**b**), and detailed view of welded chips (**c**).

Figure 4 shows the images of the Barker-etched chip-based profile. The individual chips are oriented similar, indicated by the same color in polarized light. Solely in the outer area at a diameter >8 mm very different orientations can be identified. Unlike the corresponding images of the cast-based profile (Figure 3), the grains are much more pronounced and separated from each other by clear, black interfaces, so that it can be determined that these are the interfaces of the welded chips.

For the grain structure in the chip-based profile, three different zones can be distinguished (Figure 4a). The first innermost zone is characterized by a different orientation of the individual chips. The grain boundaries correspond to the chip boundaries so that every chip contains a new grain and the grain visibility is high. Since the local strain of the chips in the center of the extrusion ram is low compared to the outer areas, the oxide break-up is insufficient, despite of the high pressure in this area during the extrusion process. This first zone is followed by a second zone in which a large number of very small, differently oriented grains can be detected in the individual chips. In the outermost zone, areas of the same grain orientation also run across the chip boundaries. This can be explained due to the process-related heat input. According to Güley et al. [11], during the extrusion process, areas of huge temperature differences can be identified in extruded profiles. Particularly in the outer areas, high energy inputs with associated temperature increases can be observed due to the frictional conditions prevailing on the die and in the dead metal zone [28]. As a result, these temperature increases lead to local exceedances of the recrystallization temperature and thus to the formation of new grains. Additionally, the local shear stress is high enough to enable recrystallization beyond chip boundaries and therefore sufficient welding of the chips, despite the local pressure decreases to zero in these regions [12]. Because of the subsequent cooling with compressed air, the cooling rates in the outer profile areas are higher. According to Liang et al. [29] higher cooling rates lead to the formation of smaller grains due to the high undercooling achieved.

The elongated grains in the chip-based profile are a result of incomplete recrystallization. Compared to the cast-based material the input of process heat is decreased because the chips are not welded at all. For this reason, the recrystallization temperature in the middle of the profile is not exceeded. In outer areas, the temperature then exceeds the recrystallization temperature, resulting in the formation of sub-grain boundaries within the individual chips. In areas further out, the temperature is then sufficiently high to exceed the recrystallization temperature and thus to cause the formation of new grains, even beyond the chip boundaries.

As already stated, Güley et al. [11] identified two critical parameters influencing the welding process of the chips. The first parameter regards to a critical shear stress above which the encasing oxide layers break down and enable metal-to-metal contact in consequence. As the second parameter, a critical path length is defined, which is understood as a minimum length of the contact of the surface of the chip surfaces in the process which is necessary to allow sufficient welding. Only if both conditions are met sufficient, a successful welding process can be achieved during the extrusion process. At least the parameter of the critical shear stress is directly influenced by the extrusion ratio. Thus, with a larger extrusion ratio, the effective shear stress is increased [14,15]. Because of the friction in the contact area between the profile and the die, the shear stress is significantly higher in the outer regions of the profile than in the central regions. For flat face dies, Haase et al. [14] were able to show that chip delamination phenomenon occurs at a lower extrusion ratio than 17.4. For the chip-based profiles, the extrusion ratio of R_p_ = 30.25 appears to be sufficient for adequate welding of the chips. Apparently, because of the effective shear stresses in the outer regions of the chip-based profile there is sufficient welding, which is why no delaminations can be found in these regions. Starting from a critical radius, the influence of the friction between the material and the die has then dropped to such an extent that sufficiently high shear stresses can no longer act to break up the oxide layers, resulting in the observed high visibility of the chips.

### 3.2. Results of Tensile Tests

The results of the tensile tests, summarized in Table 3, clearly show differences between cast- and chip-based specimens. While the tensile tests performed on cast-based specimens show a lower scattering, the chip-based specimens differ more in the results with regard to ultimate tensile strength and yield strength. The cast-based specimens have both a higher ultimate tensile strength and a higher elongation at break than the chip-based specimens. On the other hand, the cast-based specimens show lower values for the yield strength than the chip-based specimens. The lower ultimate tensile strength of the chip-based specimens can be explained by the insufficient quality of the weld seams. In addition to the reduction of the strength caused by the weld seams, the defects in the specimens in the form of delaminations also reduce the strength due to their notch effect. In general, there are clear differences between the defect sizes in the chip-based specimens which explain the higher scattering of the quasistatic properties.

The higher 0.2%-yield strength can be explained by the hardening characteristic of the material. Many investigations indicate a pronounced cyclic hardening of AW6060 [30,31]. Thus, when extruding the cast-based material already at the beginning of the extrusion process a material cohesion is given so that no additional deformations of the material are required. Regarding the chip-based material, on the other hand, material cohesion has to be created by local forming of the chips. In this way, the chip-based material has already experienced a significantly higher deformation and thus strain hardening compared to the cast-based material after the extrusion.

In order to investigate the damage mechanisms in case of tensile load, CT analyses of the specimens tested in tensile test were performed. The parameters of the tensile tests were chosen based on the force drop in such a way that the specimens do not fail completely during the test.

**Figure 5:**
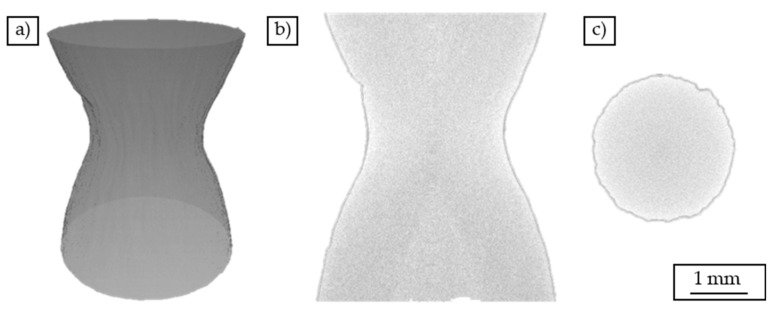
Volume reconstruction of cast-based specimen tested in a tensile test: (**a**) three-dimensional representation, (**b**) cross-sectional view parallel to the load direction, (**c**) cross-sectional view perpendicular to the load direction.

The volume reconstruction of a cast-based specimen tested in the tensile test (Figure 5) shows a significant constriction of the specimen just before the fracture. The diameter of the specimen has been reduced in this range from initially d = 7 mm to a value of d = 2.5 mm.

**Figure 6:**
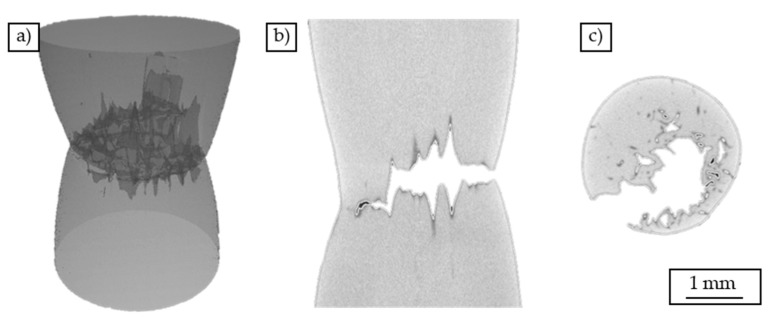
Volume reconstruction of chip-based specimen tested in a tensile test: (**a**) three-dimensional representation, (**b**) cross-sectional view parallel to the load direction, (**c**) cross-sectional view perpendicular to the load direction.

The volume reconstruction of a chip-based specimen (Figure 6) also shows a significant constriction. The diameter is reduced to a value of d = 2.7 mm. Furthermore, cracks are already visible. These propagate between individual chips and effect a separation of the material in the plane of the smallest cross-section. The cracks are rather short and located in the lower left area of the cross-section.

### 3.3. Results of Fatigue Tests

**Figure 7:**
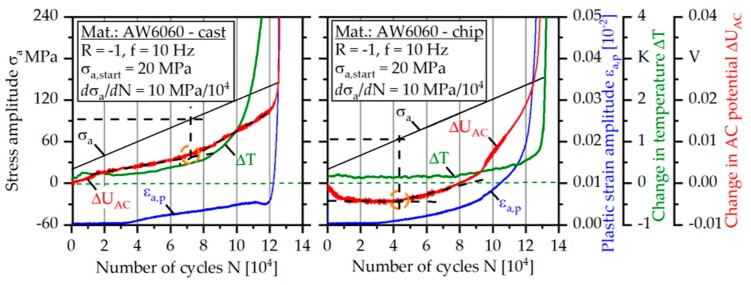
Load increase tests of cast-based and chip-based specimens.

Figure 7 shows the results of the load increase test (LIT) for the cast-based specimen as well as the chip-based specimen. 

Based on the material response caused by the continuously increasing stress amplitude, the fatigue strength, as stated in [24], can be well estimated. For this purpose, the analysis of the change in AC potential fits best, as it is the most comprehensive measurement technique. The AC potential is influenced by the temperature, the geometry of the specimen (e.g., changed because of cyclic creep), and especially the microstructure and therefore takes fatigue relevant mechanisms like dislocation accumulation and crack propagation into account [24]. As a result, the course of AC potential follows the courses of the temperature as well as the plastic strain amplitude and additionally takes microstructural changes into account, which cannot be indicated by the temperature or the plastic strain amplitude.

For the cast-based specimen two different regions of linear increase, after a short phase of initial rise of the AC potential, can be distinguished, whereby the slope changes at a stress amplitude of σ_a_ = 93 MPa. Based on the material response, the fatigue strength can be estimated at about σ_a,e_ = 93 MPa. It can be assumed that, above σ_a,e_, damage-relevant processes occur in the material, which effect the changes in the material response. The results fit well to the S-N-curve (Figure 10b), where a run out occurred in a constant amplitude test (CAT) at a stress amplitude of σ_a_ = 90 MPa.

For the chip-based specimen an initial decrease of ΔU_AC_ can be observed until σ_a_ = 35 MPa is reached due to a compaction of the weld seams presumably. In the stress amplitude region between σ_a_ = 35 and 63 MPa, the change in AC potential shows a plateau phase, followed by an exponential increase. Analogously to the cast-based specimen the fatigue strength can be estimated at the end of the first linear region at a stress amplitude of about σ_a,e_ = 63 MPa. At a stress amplitude of σ_a_ = 115 MPa, a change in the slope can be observed in the course of the plastic strain amplitude as well as in the change of the AC potential. This is due to the initiation of a second main crack on the opposite side of the first crack, which can be observed by intermittent fatigue tests for most of the chip-based specimens (Figure 9). The yield strength fits well to the stress amplitude of the first increases of the plastic strain amplitude (Table 2). The drastic increase of all measurands for the cast-based and the chip-based specimen at the end of the tests indicates the final crack propagation stage.

Based on the results of the LIT, suitable stress amplitudes for constant amplitude tests for the chip-based material were chosen. As the fatigue strength was estimated to be about 63 MPa, for reaching the high cycle fatigue (HCF)-region a stress amplitude of σ_a_ = 80 MPa was chosen. For reaching the low cycle fatigue (LCF)-region, a stress amplitude near the stress amplitude at break for the LIT was chosen (σ_a_ = 120 MPa).

**Figure 8:**
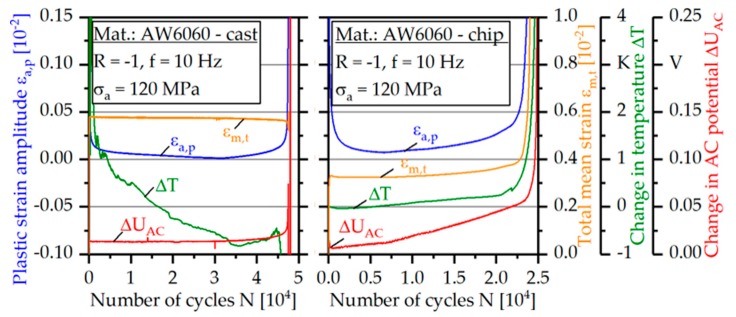
Constant amplitude tests of cast-based and chip-based specimens (σ_a_ = 120 MPa).

In the constant amplitude test with a stress amplitude of σ_a_ = 120 MPa, a significant cyclic hardening phenomenon, accompanied by a drop in the plastic strain amplitude as well as in the temperature can be recognized for the cast-based specimen (Figure 8) after an initial strong softening in the first ten cycles. In the beginning, the drop in the plastic strain amplitude runs exponentially and after about N = 5000, becomes linear. The total mean strain increases very rapidly in the first N = 50 load cycles due to a different cyclic hardening behavior in tension and compression direction.

With a number of cycles of about N = 32,000, the material starts cyclic softening, becoming exponentially until break. At the same time, an increase in the temperature is observed. The change in AC potential increases abruptly at the beginning of the test by about ΔU_AC_ = 0.015 V. Subsequently, this rises to a number of cycles of about N = 40,000 initially linear and then exponentially up to the number of cycles to failure N_f_ = 47,487.

The constant amplitude test of the chip-based specimen tested at the identical stress amplitude of σ_a_ = 120 MPa (Figure 8) shows a comparable qualitative course for the three measured measurement techniques considered. The failure occurred at a number of cycles of N_f_ = 24,180. At the beginning of the test, a strong cyclic hardening occurs up to a number of cycles of about N = 7000 detectable by a decrease of the plastic strain amplitude. Associated with this, there is a slight drop in the temperature within the first 1500 load cycles. The change in AC potential shows an even shorter reaction to the cyclic hardening with decreasing decay within the first 100 load cycles.

Subsequent to the descending course of the measured quantities, a cyclic softening of the material occurs, which is accompanied by a linear increase of all the measured quantities considered, until these change into an exponential rise until the break. The temperature measurement reacts at the earliest (from about N = 20,000) with a transition to the exponential increase, while the change in AC potential increases exponentially at the latest (from about N = 24,000).

The total mean strain clearly increases in the first N = 50 load cycles to a value of σ_m,t_ = 0.32%. After a phase of approximately constant course, this starts to increase linearly from a number of cycles of about N = 8000 linearly to a value of about σ_m,t_ = 0.37% until transition into an exponential increase at a number of cycles of about N = 22,000.

**Figure 9:**
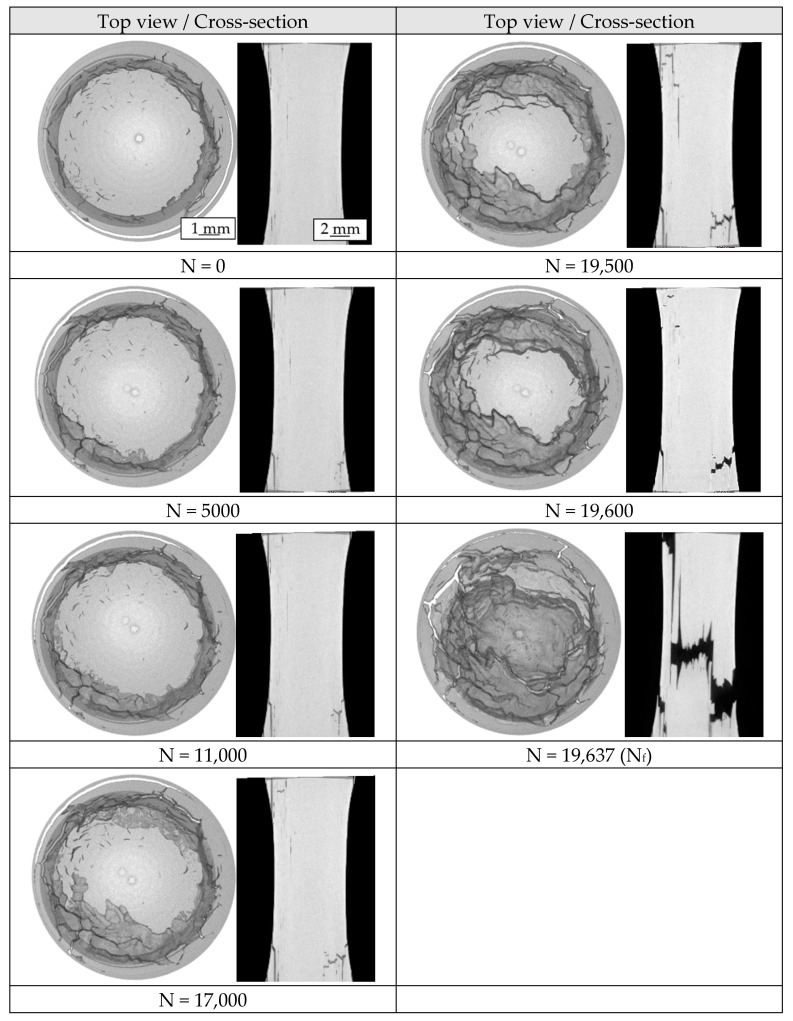
Damage development of a chip-based specimen tested in a constant amplitude test (σ_a_ = 110 MPa).

An intermittent CAT was performed at a stress amplitude of σ_a_ = 110 MPa on a chip-based specimen. CT investigations of the crack progress were performed after a certain number of load cycles. The volume reconstructions (top view) and the corresponding cross-sectional images are shown in Figure 9. In the initial state, a tubular defect due to a delamination between the chips is evident. This is because of insufficient break-up of the oxide layers due to low local strain in the innermost regions of the profile. Thereby, in micrographs chip boundaries are visible (Figure 4). The tubular defect is located in the clamping areas and in the conically extending transition area toward the gage length area. Because of the geometry of the specimen, the gage length area is located within this tubular defect, so that the test area is defect-free except of small, isolated delaminations between the chips. After N = 5000 load cycles, crack initiation and propagation can be recognized in the upper region of the specimen. Crack initiation site is the area where the tubular defect cuts the surface because of the conical shape of the specimen. After N = 11,000 load cycles, the described crack grows to a projected length of about 1.5 mm in the cross-section. Further crack initiation can already be recognized on the opposite side. With an applied number of cycles of N = 17,000 this second crack grows to a projected length of about 6 mm. In turn, the tubular defect acts as the initiation site. After N = 19,500 load cycles both cracks merge into a crack parallel to the loading direction along the chip boundaries.

The course of the change in AC potential (constant amplitude test, Figure 8) correlates well to the results of the crack propagation behavior in the intermittent fatigue test (Figure 9). From the point of discontinuity and the subsequent change of the slope, it is believed that the second crack of the chip-based specimen occurs on the opposite side of the first crack, which leads to an overlap of the crack growth rates and therefore to an increase in the slope. As can be ascertained in the intermittent experiments, crack propagation occurs already after N = 5000 load cycles. Accordingly, crack propagation already occurs at the beginning of the test, since the crack initiation phase occurring in the cast-based specimen is eliminated.

The occurring damage is correspondingly expressed in the progressive course of the change in AC potential. However, crack propagation along the grain boundaries can also be recognized for the cast-based specimens on the basis of the fractographic images (Figure 11), although the grain boundaries do not act as crack initiation sites.

**Figure 10:**
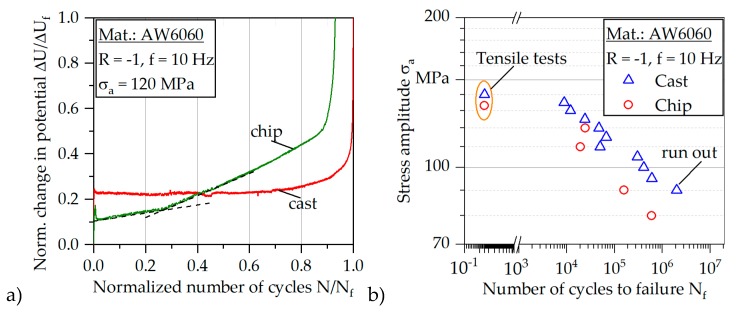
Change in potential for cast-based and chip-based specimens (**a**), S-N curve for cast-based and chip-based specimens (**b**).

In order to compare the change in AC potential, the curves of the cast- and chip-based specimens in CAT at σ_a_ = 120 MPa are shown in Figure 10a. For better comparability, both axes are normalized. While the cast-based specimen shows a constant course over a longer period of time, for the chip-based specimen a linear increase from the beginning can be recognized. The slope of this linear curve increases from about 0.3 N_f_. This can be explained with the initiation of the second crack so that both crack propagation rates summarize. The increase of U_AC_ cannot be explained by the temperature change (Figure 8), since the temperature change is not significant and also does not show a second linear increase with a change of the slope. Analogously, the increase can also not be explained by the total mean strain (Figure 8). Thus it can be clearly seen that an increase of the total mean strain by Δσ_m,t_ = 0.32% causes a change in U_AC_ of only ΔU_AC_ = 0.01 V. Accordingly, the subsequent increasing total mean strain by Δσ_m,t_ = 0.05% cannot explain the subsequent large change of the AC potential of ΔU_AC_ = 0.04 V until the beginning of the exponential increase. Since the two influencing variables of the geometry change and the temperature change can be excluded in this way, the only reason for the change in AC potential is the microstructure, especially crack propagation.

On the other hand, it remains unclear why the change in AC potential over a long period of time is linear, since, according to the Paris law [32], an increasing crack rate and thus a progressive increase should be expected. One possible explanation is given by the fiber bridging model [33]. The basis is a barrier effect of individual chip boundaries. These can act as barriers to crack propagation, so that crack propagation is prevented due to the chip boundaries. An identical phenomenon of the linear increase for fiber-based material was found in [33]. Therefore, crack propagation cannot be described using the Paris law.

Fatigue progression begins very early, as crack propagation in the intermittent fatigue test (Figure 9) shows, allowing crack growth after only N = 5000 load cycles. As already described, because of the seam welds present in the chip-based specimens, a crack initiation phase is eliminated, so that it comes directly to the crack propagation phase. In this case, the crack is apparently deflected along the individual chips. After a certain number of cycles, a second crack occurs on the opposite side due to the increase of the stress by the reduced residual area.

Based on the S-N curve (Figure 10), clear stress-dependent differences in the lifetimes of both types of specimens can be recognized. The chip-based specimens show significantly reduced lifetimes compared to the cast-based specimens. While the difference in the HCF-region is about a decade, the differences in the LCF-region are significantly lower. The outlier at σ_a_ = 110 MPa for the cast-based specimens is a result of porosity, which can be clarified by means of computed tomography. Overall, the scattering in the cast-based specimens is low. However, lifetime scattering for the chip-based specimens can be correlated with the observed variations in defect sizes.

### 3.4. Results of Fractography

**Figure 11:**
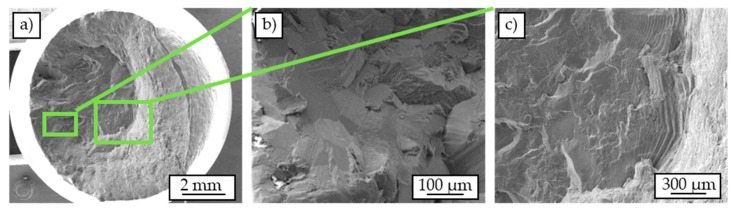
Scanning electron micrographs of a cast-based specimen tested in a constant amplitude test (σ_a_ = 120 MPa): overview (**a**), crack course along grain boundaries (**b**), striations (**c**).

The SEM-images of the cast-based specimens failed in the fatigue test (σ_a_ = 120 MPa) (Figure 11) show two characteristic areas of fatigue fracture and overload fracture which is typical for cyclically tested specimens [34,35]. The fatigue fracture area has a smooth surface covered by striations (Figure 11b). The striations increase in size toward the area of overload fracture. The fatigue crack seems to propagate along the individual grains and thus inter-crystalline. Distinct cracks can be seen between the individual grains, while the surfaces of the grains partly show striations (Figure 11c). The overload fracture shows a strong ductile deformation. Overall, the fracture mechanism resembles the cup-cone fracture mechanism described in the literature for tensile testing [35]. Preferred crack locations or crack initiation sites cannot be identified.

**Figure 12:**
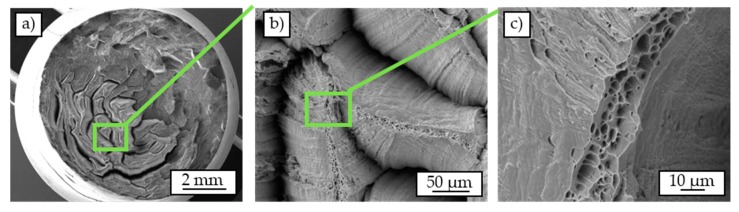
Scanning electron micrographs of a chip-based specimen tested in a constant amplitude test (σ_a_ = 120 MPa): overview (**a**), detached chips (**b**), fracture pattern on chips (**c**).

Figure 12 shows the SEM-images of a chip-based specimen (σ_a_ = 120 MPa). Compared to the cast-based specimen (Figure 11) a completely different failure mechanism can be recognized. Analogously to the cast-based specimen, two areas of different shape are apparent. There is the overload area with local separation of the individual chips (Figure 12a). This is particularly pronounced in the central regions of the material, while the outer edge regions appear crack-free in large parts. Visible in this context is the significantly reduced chip width in the peripheral areas.

In the fatigue fracture area, the chips seem hardly detached compared to the overload fracture area. Similar to the cast-based specimen (Figure 11), individual cracks propagate along the grain boundaries. Considering the individual chips with higher magnification in the first area (Figure 12c), local areas with a ductile honeycomb fracture can be recognized on almost all chip surfaces.

## 4. Conclusions and Outlook

Within the scope of this work, investigations were carried out with the aim of the mechanical characterization of directly recycled, hot extruded chip-based profiles. The following may be concluded:

(1) The microstructure of chip-based profiles is characterized by three different areas, which originate due to different recrystallization zones.
(2) While micrographs of chip-based profile did not show a delamination between the single chips, it was found that there is a critical diameter where the combination of the necessary properties of high strain and pressure is considered too low to lead to a sufficient welding, which leads to crack initiation, as shown by intermittent test strategy. Therefore, the initiation of the two separate cracks was observed. Because of the insufficient welding of the chips, the cracks propagate between the chip boundaries.
(3) The weld seams occurring between the chips have a significant influence on the mechanical properties of the resulting profiles. While the quasistatic properties are only slightly reduced by about 5%, the insufficient welded chips in the innermost area of the profile lead to a reduction of the load dependent fatigue life up to one decade.
(4) The load increase test procedure is well suited in order to estimate the fatigue strength with only one specimen.
(5) As the weld seams act as crack initiators, crack propagation phase begins very early for chip-based specimens.

In further studies, concepts of the finite element method will be used to verify by simulation, to what extent the assumption of stress concentration on the opposite side of the first crack is justified. Additionally, the fiber-bridging model will be applied in order to simulate the effects of the chip boundaries on the crack propagation behavior. The findings will then be used to develop a comprehensive material model for chip-based extrusion profiles.

## Figures and Tables

**Table 1:** Chemical analysis of AW6060 aluminum cast alloy (wt.%).

Ref.	Si	Fe	Mn	Mg	Zn	Ti	Al
**DIN EN 573-3**	0.3–0.6	0.1–0.3	<0.1	0.35–0.6	<0.15	<0.1	Bal.
**XRF**	0.4	0.21	0.04	0.42	0.01	0.01	Bal.

**Table 2:** Parameters and settings of computed tomography (CT) examinations for measurements in the gage length area of tensile and fatigue specimens.

Exposure Time	Number of Frames	Beam Intensity	Beam Current	Beam Power	Resolution
250 ms	8	135 kV	98 µA	13.2 W	13.5 µm

**Table 3:** Material characteristics obtained from tensile tests.

Characteristic Value	Cast-Based	Chip-Based
0.2%-yield strength *σ_y,__0.2_* (MPa)	45.9 ± 0.5	54.1 ± 5.4
Ultimate tensile strength *σ_UTS_* (MPa)	140.5 ± 1.7	133.3 ± 5.8
Elongation at break *ε_f_* (%)	26.6 ± 2.9	18.2 ± 0.6
